# L1 Retrotransposons: A Potential Endogenous Regulator for Schizophrenia

**DOI:** 10.3389/fgene.2022.878508

**Published:** 2022-06-27

**Authors:** Muhammad Jahangir, Li Li, Jian-Song Zhou, Bing Lang, Xiao-Ping Wang

**Affiliations:** Department of Psychiatry, National Clinical Research Center for Mental Disorders, The Second Xiangya Hospital of Central South University, Changsha, China

**Keywords:** retrotransposons, LINE-1, schizophrenia, somatic mutation, chromatin remodelling

## Abstract

The long interspersed nuclear elements 1 (LINE-1/L1s) are the only active autonomous retrotransposons found in humans which can integrate anywhere in the human genome. They can expand the genome and thus bring good or bad effects to the host cells which really depends on their integration site and associated polymorphism. LINE-1 retrotransposition has been found participating in various neurological disorders such as autism spectrum disorder, Alzheimer’s disease, major depression disorder, post-traumatic stress disorder and schizophrenia. Despite the recent progress, the roles and pathological mechanism of LINE-1 retrotransposition in schizophrenia and its heritable risks, particularly, contribution to “missing heritability” are yet to be determined. Therefore, this review focuses on the potentially etiological roles of L1s in the development of schizophrenia, possible therapeutic choices and unaddressed questions in order to shed lights on the future research.

## Introduction

Retrotransposons are a group of “jumping genes” which constitute ∼17% of the human genome. In fact, nearly half of the human genome (∼45%) is derived from insertions of transposable elements (Lander et al., 2001). The mechanism and further details of retrotransposons have already been extensively reviewed elsewhere (Terry and Devine, 2020). Retrotransposons are divided into LTR or non-LTR retrotransposons with the presence or absence of long terminal repeat sequences (LTR), respectively. LTR retrotransposons are also known as endogenous retroviruses (ERVs) due to similar mechanism and structure to simple retroviruses (Garcia-Perez et al., 2016). Non-LTR RTEs (Retrotransposable elements), are further classified into autonomous RTEs LINEs (Long interspersed nuclear elements) and Non-autonomous RTEs (Alu) (Lander et al., 2001). Non-autonomous non-LTR retrotransposons are collectively known as SINEs (short interspersed elements) and there are also SVA SINEs in humans, in addition to Alu (Ostertag et al., 2003; Wang et al., 2005). All these actively promote genetic diversity, mutations as well as human diseases (Lander et al., 2001). Although there are three major LINE families (L1, L2, and L3), only the L1 family can transpose in the human genome (Moran et al., 1996). L1 performs reverse transcription at the genomic target site, in a process known as target-primed reverse transcription (Luan et al., 1993). LINE-1 (L1) mediated insertions are regarded to be a kind of endogenous mutations capable of causing deletions/insertions and copy number variation which are all well-established risk factors for neurological disorders including Alzheimer’s disease, autism and schizophrenia (Baillie et al., 2011; Guffanti et al., 2014). Increased level of L1 copy number has also been reported in the blood of animal models, major depressive disorder (MDD) patients (Liu et al., 2016) and post-traumatic stress disorder (PTSD) subjects due to hypomethylation (Rusiecki et al., 2012). Discussing all aspects of transposable elements and their consequences on genome health for each disorder is beyond the scope of this article, therefore, this review is however mainly focused on L1 and its involvement in schizophrenia development.

### L1 Retrotransposition in Brain Cells

Retrotransposable elements (RTEs) are only known to be de-repressed in the brain during human life. Accumulation of approximately 13.7 novel somatic L1 insertions have been noticed in human hippocampus (Upton et al., 2015). Although this number remains arguably controversial (Sanchez-Luque et al., 2019), the consensus conclusion is that mature neuronal cells support somatic L1 retrotransposition which has been evidenced in non-dividing neurons *via* engineered L1 retrotransposition (MacIa et al., 2017). Furthermore, L1 mis-regulation in brain tissues has been found associated to neurological diseases, and the putative reasons may be: 1) Increased RTE expression/activity due to mutations of RTEs-regulating genes, 2) genetic and environmental components and 3) time-dependent accumulation of L1 copy number, neuronal degeneration and phenotypes associated with aging (Terry and Devine, 2020).

L1s’ mobilization occurs more frequently during differentiation of neurons than non-neuronal cell types (Coufal et al., 2009). However, L1s can also be mobilized in postmitotic neurons (MacIa et al., 2017). Likewise, the rat hippocampus also presents L1 retrotransposition activity during adult neurogenesis, indicating a strong retrotransposition activity in neural progenitor cells even at adult stage (Muotri et al., 2009). L1 copy numbers are also significantly higher in various areas of healthy adult human brains (especially the hippocampus) when compared to the liver and heart of the same person (Coufal et al., 2009; Upton et al., 2015). Terry et al. have proposed that in the context of findings by Muotri and Coufal et al., somatic L1 retrotransposition seems to occur at all phases of neuronal life, including mature or developing neurons, differentiating neural stem cells and neuronal progenitor cells (Coufal et al., 2009; Muotri et al., 2009; Terry and Devine, 2020). In light of these aforementioned studies, it is assumed that probably the L1 frequency is associated with cellular active engagement into neuronal circuits. Cells with more activity may have higher retrotransposition rate. However, many questions still remain unaddressed. For example, is L1 associated with increased pyramidal cell activity or reduced activity of inhibitory neurons? Is retrotransposition active at specific time window only or throughout the life span of a cell? If it is active throughout the life, then which stage of life is associated with the harmful effects? Studies using proper animal models would help to answer these questions.

Most L1s integrate into non-exonic regions and won’t cause any recognized phenotypes. So far, no hotspots for L1 insertion have been discovered in the genome. The question of whether L1 insertion is random or guided by environmental factors, hormone influence, or inherited genetic print remains unanswered.

### L1 Retrotransposition, a Putative Risk Factor of Schizophrenia

The insertion of L1 has long been proposed to predispose people with the risk of schizophrenia (Doyle et al., 2017). The significant increase in copy number of L1 has been confirmed in the postmortem prefrontal cortex of schizophrenia patients (Bundo et al., 2014). In addition, L1 copy number in neurons was markedly increased in contrast to non-neuronal cells in schizophrenia patients (Bundo et al., 2014). Moreover, genomic analyses of brain tissues from animal models which utilized poly I:C and chronic epidermal growth factor to produce schizophrenia-like phenotypes also revealed an increase of L1 copy number, implying the impact of prenatal and postnatal stress (Bundo et al., 2014). Antipsychotics have no influence on L1 copy numbers. Moreover, consistent increase of L1 copy number has been observed in iPS cell-derived neurons of schizophrenia patients with 22q11 deletion (Bundo et al., 2014). This suggests that a well-defined substantial genetic risk factor indeed contributes to the concentration of L1 in the brain (Bundo et al., 2014). Moreover, Whole Genome-Sequencing (WGS) data have suggested that L1s preferentially insert into genes related to synaptic functions (Bundo et al., 2014; Doyle et al., 2017). Baillie et al. also determined that L1s are mostly enriched in genes responsible for the neuronal synapse, axogenesis, postsynaptic density and presynaptic membrane (Baillie et al., 2011; Bundo et al., 2014; Doyle et al., 2017), which indicates the L1 retrotransposition specifically affects activity at neuropil. However, the exact reason for possible integration of L1s into synaptic genes is unknown.

It is worth noting that the retrotransposition itself can cause many by-products which may have detrimental consequences. For example, because of its endonuclease activity, the ORF2p product of L1 might generate mutations and instability. The expression of faulty protein, RNA or DNA in the cytosol may also trigger immune response, inflammation and neuron degeneration (Terry and Devine, 2020). Dysregulated expression of retrotransposable elements (RTEs) can be extremely harmful for a number of reasons. First, high levels of RTE proteins, RNAs, or extrachromosomal cDNA copies can cause cellular toxicity and activate inflammatory response pathways. Second, such expression allows for functional RTE replication, which could result in insertional mutagenesis, activation of the DNA damage response, or even programmed cell death (Dubnau, 2018). Immune activation models simulating both viral infection and inflammation have been used to investigate possible links between perinatal environmental risk factors for schizophrenia and L1 activity. In both the mouse and macaque models, an increased L1 copy number in the brain was observed in response to these two perturbations, indicating that the L1 content in the brain is likely influenced by early environmental factors (Bundo et al., 2014). Although L1 mobilisation can occur during neurogenesis as well as later stages of neuronal development which eventually leads to individual somatic mosaicism (Lupski, 2013; McConnell et al., 2013; Poduri et al., 2013), it remains unaddressed why the schizophrenia symptoms appear later in life. Is there any difference between L1 retrotransposition pattern and frequency at early and adult developmental stages? Is there any difference in L1s frequency between inhibitory and excitatory neurons? Moreover, genes responsible for synaptogenesis have been reported dysfunctional in schizophrenia (Gandal et al., 2018). Do L1s also integrate into the open reading frame of synaptogenesis genes? Do L1s induce the disruption of synapse-forming genes alone or in combination with other stimuli?. Nevertheless, it is uncertain how the L1s target precisely at those genes. Are there specific sequences in genes for synaptogenesis, which are sensitive towards L1s? Or a parallel protein is transcribed along with L1s which is specific for some nucleotide sequences and exclusively recruits L1s towards genes for synaptogenesis? Though L1 is emerging as a possible cause of schizophrenia, it may be equally possible that L1 retrotransposition may be the pathological consequence of schizophrenia. These questions remain largely unclear and shall be warranted for further study. Moreover, Bundo et al., 2014 have studied L1 retrotransposition in multifaceted clinical settings; Postmortem brain tissue-iPSCs-animal model. However, further clinical studies are essential to determine the unexplored aspects of L1 and schizophrenia molecular biology from real biological environment (particularly postmortem studies) to provide additional evidence.

### L1, Interferons and Schizophrenia

Activation of endonuclease-dependent L1 retrotransposon can increase the expression of endogenous IFN-β and IFN-stimulated genes which in-turn suppress L1 propagation (Yu et al., 2015). *In vitro* study also suggested that endogenous IFN signaling limits the propagation of L1 retrotransposon. Collectively it is suggested that IFN may play a protective role against L1 retrotransposon activation and propagation. The activation of L1 possibly activates the expression of low levels of IFN, which in turn antagonize the subsequent L1 retrotransposition. This hypothesis is supported by a correlation between L1 and IFN-β mRNA expression and the capacity of exogenous L1 to induce the expression of IFN-β and downstream substrates in vitro (Yu et al., 2015). It is not clearly understood whether INF-β can be harnessed as a therapeutic option for schizophrenia. Excessive accumulation of L1 DNA in the cytosol of neurons [due to three-prime repair exonuclease I (TREX1) deficiency] can precipitate type 1 interferon (IFN-1) inflammatory response and subsequent apoptosis (Thomas et al., 2017). The response of IFN-1 could be ameliorated by inhibitors of the L1 reverse transcriptase, implicating that L1 reverse transcriptase is an appropriate target for the treatment (De Cecco et al., 2019). But questions still remain unanswered. For example, which and when L1 should be inhibited? Cognitive symptoms often precedes psychosis (Mintz and Kopelowicz, 2007), therefore it is intriguing whether administration of L1 reverse transcriptase inhibitor in cognitively impaired mice would provide more mechanistic insights for schizophrenia. Nevertheless, negative correlation between IFN-γ and cognition in patients with schizophrenia has been reported recently (Wilson et al., 2018).

**FIGURE 1:**
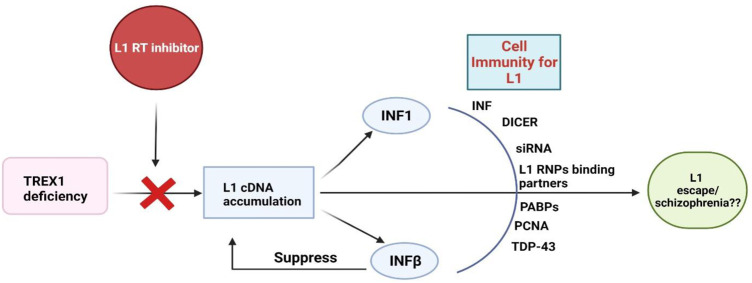
Cellular immune responses to L1s. **TREX1:** Three Prime Repair Exonuclease 1, **INFs**: Interferons, **PABPs:** poly-A binding proteins, **PCNA:** Proliferating cell nuclear antigen, **TDP-43:** TAR DNA binding protein-43.

Whilst L1s exploit the cellular machinery to achieve replication, the host cells also have developed a number of defense mechanisms to counteract L1 toxicity. Innate cellular immunity and inhibitory elements for L1 retrotransposition include IFNs, RNA mediated regulation, post transcriptional silencing *via* DICER and siRNA, L1 RNPs binding partners, Poly A binding proteins (PABPs), PCNA and other regulatory elements have been extensively elaborated somewhere else (Pizarro and Cristofari, 2016). TAR DNA binding protein 43 (TDP-43) is a protein which binds with the RNA transcript of L1. Mutated TDP-43 presents reduced binding with L1 RNA which in turn results in elevated L1 transcripts (Figure 1) (Li et al., 2012). Nevertheless, the exact inhibitory mechanism and associated elements contributing to the process are not fully understood. Of note, despite of the cell’s precise mechanisms for regulating transposable elements (TEs) activity, certain TEs are still able to escape repression and produce new integration in germ cells during early embryonic development and in somatic tissues later in life (Baillie et al., 2011; Kazazian, 2011; Lee et al., 2012).

The interaction of L1-IFNs-schizophrenia pathology is still vague and not explored enough. For example, how does L1 trigger INF response and how INFs fail to respond to L1 and drive schizophrenia remain entirely unclear. Do the truncated L1 transcripts or their translated protein accumulation causes inflammatory response/toxicity and ultimately serves as a cause of schizophrenia development? How does the L1 escape the regulation of IFN? Does the escape occur in a cell/time specific manner? Do the IFNs only work against truncated L1 or also the full length L1s? Further research is warranted to address these questions.

### L1s and Somatic Mutations

The L1 elements are non-LTR transposons which can transpose in neural progenitor cells during brain development and might contribute to intra-individual difference in brain function (Coufal et al., 2009; Evrony et al., 2012; Erwin et al., 2016). Single cell sequencing and genome wide analysis confirmed few L1 somatic insertions in normal human caudate and cortical neurons, which however argues that L1 retrotransposition is the main source of neuronal diversity in the human cerebral cortex and caudate nucleus (Evrony et al., 2012). Human specific L1 (L1Hs) elements integrate favorably into genes linked with neuronal functions and diseases (Jacob-Hirsch et al., 2018). For example, the DLG2 gene, which is frequently mutated in schizophrenia, had 141 somatic insertions of L1 in the brain samples (Jacob-Hirsch et al., 2018) and thus was considered as a target for somatic L1-associated variants (Erwin et al., 2016). In brain tissues, the number of retrotranspositions is higher than non-brain samples, and even higher in brains suffering from tuberous sclerosis complex, Rett syndrome, ataxia-telangiectasia or non-syndromic autism (Jacob-Hirsch et al., 2018). Most of somatic brain retrotransposons incorporate into pre-existing repetitive elements, favorably A/T-rich L1 sequences, and form nested insertions. Those pre-existing retrotransposons may serve as “lightning rods” for new insertions, which allows deliberately-regulated gene expression in order to safeguard detrimental outcome. Therefore, the dysregulated retrotransposition may compromise this safety mechanism and increase the likelihood of detrimental mutagenesis in neurodevelopmental diseases (Jacob-Hirsch et al., 2018). However, the mechanism for selective target site insertion of L1 is not known and remains open for debate, for example, which L1s prefer nested insertion and which one goes for harmful mutagenesis predisposing to the development of schizophrenia? Moreover, the utilization of single cell sequencing approach will not only assist to study the role of L1 retrotransposition, but the different behaviors and functions of various cell types in context of their microenvironment which may have impact on L1 retrotransposition and schizophrenia.

The postzygotic somatic mutations (PZMs), which include epimutations (changes in histone modifications and promoter methylation that affect gene expression but not the DNA sequence), usually result in somatic mosaicism. Compared to other tissues, the PZMs are comparatively common in mammalian brains especially in schizophrenia patients (Singh et al., 2020). Moreover, It has been found that harmful somatic mutations found in schizophrenia brains were enriched in schizophrenia-related pathways including dopaminergic and glutamatergic pathways or long-term potentiation process (Kim et al., 2021). The brain somatic mutations, particularly in GRIN2B (one of the subunit of NMDA receptor), disrupt the localization of GluN2B to dendrites and impair proper synapse formation (Kim et al., 2021).

### L1 Hypomethylation and Schizophrenia

The promoter region of DNA regulates gene transcription and its methylation shields the binding of transcription factors, ultimately silencing the gene expression. Hypomethylation exposes promoter to transcription factors and allows the subsequent transcription or protein expression. In mammalian genomes, L1 is the active autonomous retrotransposon, and hypomethylation of L1 is associated with higher retrotransposon activity. Analysis of peripheral blood samples revealed significant hypomethylation of L1 in schizophrenia patients (Misiak et al., 2015; Liu et al., 2016) both in first episode schizophrenia and chronic schizophrenia (Murata et al., 2020). However, it is intriguing whether the L1 methylation in peripheral blood is truly indicative of L1 methylation in the brain. Hypomethylation of L1 has also been noticed in other mental disorders like MDD and PTSD (Saurez et al., 2018). It is not yet clear whether the pattern of L1 retrotransposition in schizophrenia is similar with or different from other psychiatric disorders. However, DNA methylation is reported variable to adapt to neuronal activity alteration (Guo et al., 2011) and it could likely mediate or contribute to the integration of environmental stimuli into diseased cell features, resulting in neuronal dysfunction (Linde and Zimmer-Bensch, 2020).

Paradoxically, hypermethylation of L1 in brain tissue of schizophrenia patients has also been reported (Fachim et al., 2018), which indicates a globally elevated DNA methylation in schizophrenia. DNA-methyltransferases (DNMTs) help to establish the DNA methylation pattern since embryonic stages and up-regulated DNMTs have been detected in the brains with schizophrenia (Grayson et al., 2006; Zhubi et al., 2009). However, each variant of DNMTs may contribute in different capacity to the onset of schizophrenia. Recently, it was reported that DNMT3B rs2424932 was strongly associated with gender and DNMT3B rs1569686 associated early age onset of schizophrenia while DNMT3L rs2070565 associated with family history and early onset of schizophrenia. Altered activity of DNMTs indeed suggests that the genetic nature of methyltransferases should be taken into account when dealing with epigenetic events mediated by methylation in schizophrenia, (Saradalekshmi et al., 2014). In human neural progenitor cells (hNPCs), deletion of DNMT1 results in hominoid-specific L1’s transcriptional activation and chromatin remodeling. The activated L1s act as alternate promoter for several neuronal protein-coding genes affecting neuronal functions, suggesting a hominoid-specific L1-based transcriptional network influenced by DNA methylation that influences neuronal protein-coding genes (Jönsson et al., 2019).

DNA methylation is generally thought to hamper the binding of transcription factors through the action of methyl-CpG-binding domain proteins, thus considered a repressive epigenetic feature (Curradi et al., 2002). The L1 promoters are C–phosphate–G (CpG) rich regions and are highly methylated and silenced under normal conditions (Steinhoff and Schulz, 2004). Because non-LTR retrotransposons encompass one-third of all CpG sites in humans (Cordaux and Batzer, 2009), silencing L1 expression *via* CpG DNA methylation and histones modification is a key repressive mechanism preventing mutagenic events from accumulation (Bourc’his and Bestor, 2004; Castro-Diaz et al., 2014; Jacobs et al., 2014). A robust molecular tool, dCas9-MQ1^Q147L^ system, has been recently developed to introduce *in vivo* site-specific DNA methylation editing with high specificity and activity (Lei et al., 2017). Although dCas9-MQ1^Q147L^ has not been tested in schizophrenia pathology, it has opened a window towards *in vivo* methylome editing and personalized medicine to alleviate the disease phenotype. Further improvement of the technique and skills are required to refine the editing efficiency.

Methyl CpG binding protein (MeCP2) is a protein that plays a role in global DNA methylation as well as neurodevelopmental disorders. MeCP2 appears to be crucial for normal functioning of nerve cells and serve as an inhibitory factor for L1 retrotransposition (Muotri et al., 2010). L1 retrotransposition can be manipulated in a tissue-specific fashion, and disease-related genetic alterations can affect neuronal L1 retrotransposition frequency (Muotri et al., 2010). DNA methylation may suppress L1 production in neural stem cells by attracting MeCP2, as evidenced by a group of CpG sites within the L1 promoter that showed a tendency to de-methylate during neuronal differentiation (Muotri et al., 2010).

### L1, Chromatin Remodeling, and Schizophrenia

The structural alteration of histone proteins within the nucleosome mediates transitions between euchromatin and heterochromatin, which are associated with active and inactive transcription respectively (Grayson and Guidotti, 2013). DNA methylation can target retrotransposons and result in a repressive chromatin conformation that can access and silence the coding sequences in their proximity (Singer et al., 2010). Insertions of retrotransposons into open chromatin (Euchromatin) is assisted, whereas, insertions into condensed chromatin (heterochromatin) is unlikely. Another possibility is that L1 inserts randomly in accessible chromatin, impacting many genes and producing a wide range of transcriptional alterations (Singer et al., 2010). It is speculated that *de novo* L1 insertions selectively target CpG-poor promoters of NPC-specific genes because L1 endonuclease identifies an A+T-rich sequence motif. In fact, insertions into A+T-rich introns of housekeeping genes are equally possible (Singer et al., 2010). A recent report has proposed that pre-existing L1s within A/T rich sequences in the genome may serve as lightning rods for retrotransposons and support nested insertion to avoid harmful mutations (Jacob-Hirsch et al., 2018).

Chronic drug treatment may activate certain intergenic repetitive sequences, eventually leading to abnormally expressed retrotransposable elements. It has been demonstrated that repeated cocaine administration decreases histone H3 Lys9 trimethylation (H3K9me3) binding and activates several specific retrotransposons: L1, SINEs, and LTRs) but had dramatically increased L1 expression in NAc of the brain (Maze et al., 2011). Cocaine may potentially have nonspecific or random effects on H3K9me3 enrichment across the genome, with inconsistent or little effect on neuronal function (Maze et al., 2011). It is speculated that each environmental stimulus may have variable effects on brain regions. However, how does the cocaine-chromatin interaction precipitate the onset of schizophrenia is unclear, although histone modifications, DNA methylation and chromatin structure in schizophrenia has been detailed (Abdolmaleky and Thiagalingam, 2014; Duan, 2019).

**FIGURE 2:**
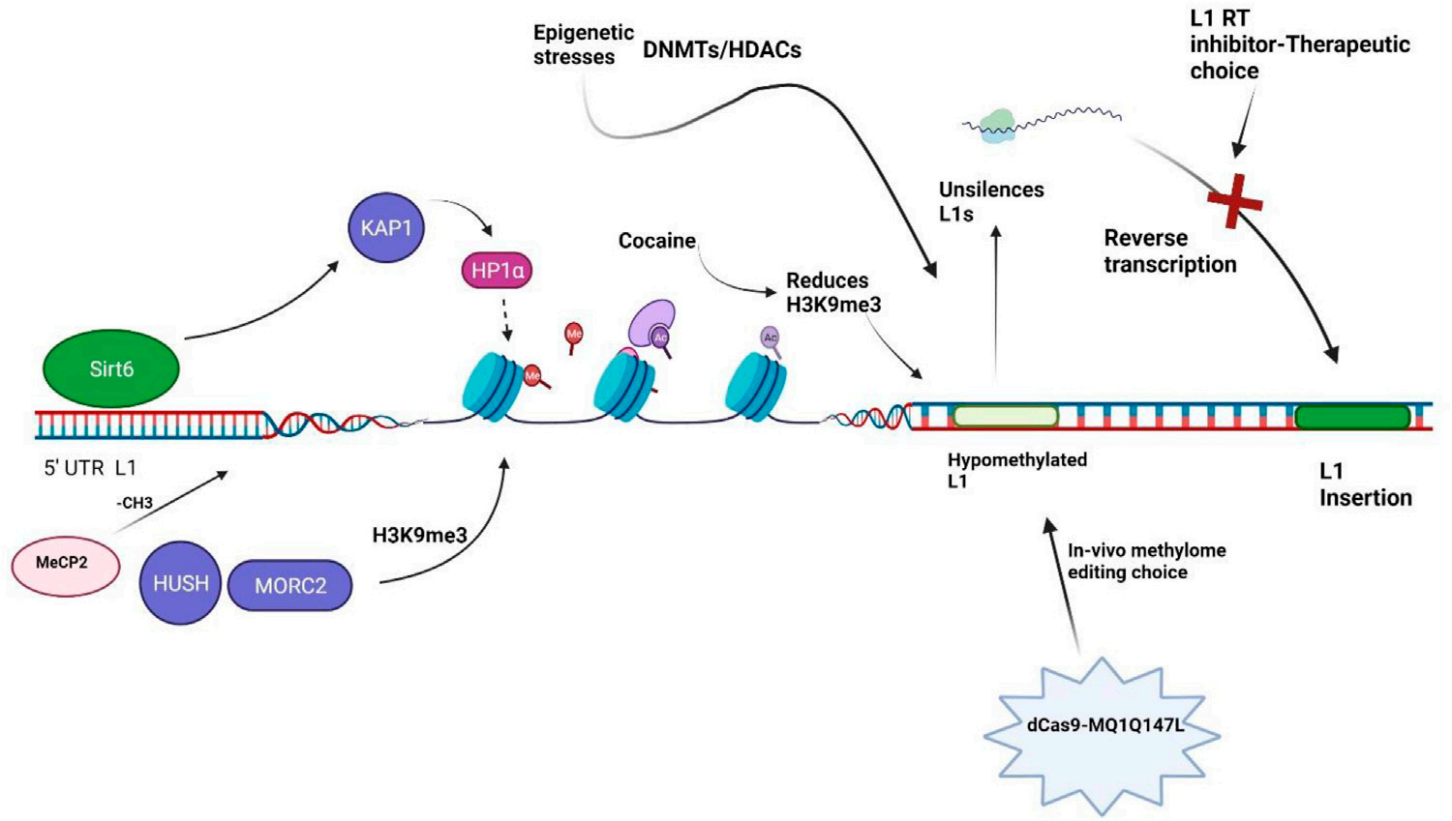
Illustration of chromatin remodelers. **Sirt6:** Sirtuin 6, **KAP1:** Krüppel-associated box 1/KRAB-associated protein 1, **HP1α:** Heterochromatin Protein 1α, **H3K9me3:** methylation of lysine 9 of histone H3, **HUSH:** human silencing hub-complex, **MORC2:** Microrchidia CW-type Zinc Finger 2, **DNMTs:** DNA methyltransferases, **HDACs:** Histone Deacetylases, **MeCP2**: Methyl-CpG-binding protein 2, **L1 RT**: L1 reverse transcriptase.

It is reported that SIRT6 is a potent suppressor of L1 retrotransposon activity (Van Meter et al., 2014). SIRT6 binds to the 5′-UTR of L1 loci, where its mono-ADP ribosylates the nuclear corepressor protein KAP1 and assists its interaction with the heterochromatin factor HP1α. HP1α then contributes to the packaging of L1 elements into transcriptionally suppressed heterochromatin (Figure 2). Depletion of SIRT6 from L1 loci in response to DNA damage allows previously silenced retroelements re-activated (Van Meter et al., 2014). But this mechanism has not been investigated in the context of schizophrenia biology.

In neurodevelopmental disorder cohorts, mutations in genes which encode chromatin remodelers are overrepresented (Mossink et al., 2021). Chromatin remodelers impacts the growth, migration, and circuit integration of cortical cells including GABAergic/glutamatergic neurons and glia (Mossink et al., 2021). A study has showed that GAD67 mRNA levels are lower in schizophrenia patients, and the amount of methylation at the associated promoter in these patients is significantly lower in the repressive chromatin fraction (Huang and Akbarian, 2007). However, the effects of chromatin remodeling in individual brain cell type and its association with L1 retrotransposition or schizophrenia is still a loop which needs further investigation. Chromodomain helicase DNA binding protein 2 (CHD2) gene is involved in neurogenesis, chromatin remodeling, and gene expression, and detrimental mutation in CHD2 has been found relevant to the onset of schizophrenia in children (Poisson et al., 2020). However, the precise roles of CHD2 in L1 retrotransposition is yet to be established, which needs further investigation to delineate the mechanism for the treatment of schizophrenia.

A study in cancer cells has identified 142 genes capable of activating or repressing L1 retrotransposition (Liu et al., 2018). These genes, which are widely associated to human diseases, regulate the life cycle of L1 at the transcriptional or post-transcriptional level, depending on the endogenous L1 nucleotide sequence, highlighting the intricacy of L1 regulation. HUSH and MORC2 preferentially bind evolutionarily young, full-length L1s in in euchromatic setting which is transcriptionally permissive, and trigger the deposition of histone H3 Lys9 trimethylation (H3K9me3), which silences transcription (Figure 2) (Liu et al., 2018). Of note, these silencing events usually occur inside introns of transcriptionally active genes, resulting in HUSH, MORC2, and L1-dependent downregulation of host gene expression. This is an excellent example illustrating how epigenetic silencing of transposable elements helps to rewire host gene expression programs (Liu et al., 2018). HUSH and MORC2 work together to preferentially target young, full-length L1s in transcriptionally permissive euchromatic areas (Liu et al., 2018). These L1s are actually the greatest threat to genomic stability and integrity, as a subclass of them remains active and mobile (Liu et al., 2018). The HUSH- and MORC2-dependent L1 silencing mechanism needs to be explored in the schizophrenia patient-derived cells. It is intriguing whether the HUSH-MORC2 molecular apparatus can be harnessed to rescue the L1 insertion in genes for better treatment of schizophrenia.

### Missing Heritability, Schizophrenia and L1s

As opposed to more common disorders such as anxiety or depression (Sullivan et al., 2013; Meier et al., 2019), schizophrenia has much higher heritability estimates of the genetic components (Ripke et al., 2014) Previous GWAS study has estimated approximately 23% of the variance in liability in schizophrenia (Blanco-Gómez et al., 2016; Maroilley and Tarailo-Graovac, 2019). This is in marked difference to results from family studies, which show that heritability for schizophrenia accounts for 60–80% of disease risk, and even beyond 80% in twin studies (Sullivan et al., 2003; Avramopoulos, 2018). However, the current data from genetic analysis does not directly corroborate the heritability estimates, resulting in the so-called “heritability gap” in psychiatry (van Calker and Serchov, 2021). Furthermore, the majority of genetic alterations identified by GWAS studies falls into non-coding regions of DNA (intergenic regions and introns) (Welter et al., 2014), which makes it difficult to validate their potential pathogenic roles in psychiatric illnesses.

It has been proposed that transposable elements contribute in genome expansion and modification not just through transposition but also through the generation of tandem repeats (Ahmed and Liang, 2012) and tandem repeats contribute in schizophrenia pathology (Grube et al., 2011). The KCNN3 is a schizophrenia potential risk gene that encodes a small conductance calcium-activated potassium channel (SK3) that regulates neuronal firing patterns. It has been reported that the short tandem repeats (STR) affects the SK3 potassium channel function and the cognition of schizophrenia patients (Xiao et al., 2021). Longer CAG repeats can reduce the SK3 channel activity in transfected HEK293 cells, which was consistent with the dysfunctional higher cognitive abilities caused by SK3 overexpression in animals (Grube et al., 2011; Martin et al., 2017). However, it should be noted that tandem repeats are typically multiallelic (Nithianantharajah and Hannan, 2007) which makes them difficult to genotype using SNP-based GWAS array platforms. This may contribute greatly to the “missing heredity” of psychiatric disorders (Xiao et al., 2021). In line with this, Kuhn et al. have suggested that SNP array-based GWAS studies would have overlooked possible phenotypic impacts of L1s (Kuhn et al., 2014), and that L1s may play a role in the “missing heritability” (Manolio et al., 2009). Though, the transposable elements contribute in generating tandem repeats but L1s specific contribution in generation of tandem repeats and eventually to “missing heritability” is the unexplored aspect, and further study will assist to find out the unseen heritable risks and their mechanism of development consequencing into schizophrenia.

### Animal Studies

Many studies using mouse models have revealed that joint exposure to peripubertal stress and prenatal immune challenge induces synergistically pathological effects on neurochemistry and adult behavior. The offspring of poly-I:C mouse model had worsened schizophrenia-like phenotypes, if subjected to environmental stress in puberty, signifying that early environmental stress can lower the threshold for the onset of schizophrenia (Giovanoli et al., 2013). That is to say, the environmental stress can increase the frequency of L1 insertions and increase the susceptibility to schizophrenia, probably *via* altered expression of synaptic or other schizophrenia-related genes in neurons (Bundo et al., 2014). Several environmental factors, such as alcohol and cocaine consumption (Maze et al., 2011; Ponomarev et al., 2012), stress (Ponomarev et al., 2010; Hunter et al., 2012) and exercise (Muotri et al., 2009) have been confirmed to alter L1 expression in the adult brain. Early life experience such as maternal care overrides the activity of L1 in mice and alters DNA methylation (Bedrosian et al., 2018), implicating early life bereavement or stress could increase the occurrence of L1 retrotransposition. Further investigation is needed to determine whether the L1 activity in early life depends on the severity of environmental stimuli or duration of exposure. A recent study revealed that L1 retrotransposition is up-regulated in the adult hippocampus after novel exploration (Bachiller et al., 2017). This shows that activities of L1 retrotransposition may underpin hippocampal activation-based memory formation in the adult brain (Bachiller et al., 2017). Consistently, Coufal and colleagues also discovered an enrichment of L1 ORF2 copy number in the adult mouse hippocampus compared to other brain areas. Furthermore, the activity of engineered human L1 retrotransposition can be detected in neural progenitor cells derived from human embryonic stem cells or isolated from human fetal brain, demonstrating that L1 components might be activated as early as the formation of the central nervous system (Coufal et al., 2009). More than one third of non-reference L1s are found within the open reading frames of protein-coding genes implicated to schizophrenia (Guffanti et al., 2016) which strongly suggests the close association between L1 retrotransposition and schizophrenia (Saurez et al., 2018).

It has been evidenced that L1 retrotransposition is required for physiological neuronal activity during memory formation in the hippocampus. L1 can affect memory formation in a time-dependent manner, specifically the Long-term memory (LTM) (Bachiller et al., 2017) which is largely based on the functional strengthening of existing synapses as well as the formation of new synapses (Radwanska et al., 2011). Intriguingly, L1 is specifically enriched in genes responsible for synaptic function in schizophrenia (Baillie et al., 2011; Bundo et al., 2014; Doyle et al., 2017). How L1 contributes to memory formation during novel exploration and the onset of schizophrenia is not clear. Probably two classes of L1s exist: “good” and “Bad” L1s, based on temporal activation, integration site or L1 guiding elements. There is also a scope for developing poly I:C animal model to check time dependent L1s profile which could be intervened *via* siRNA capable of crossing Blood-brain barrier. It is also needed to explore whether the pattern of L1 retrotransposition is heritable.

Furthermore, only ∼ 100 retrotransposition-competent (RC) different classes of L1s are found in individual human (Brouha et al., 2003; Beck et al., 2010) whereas, approximately 3000 RC L1s are found in mice (DeBerardinis et al., 1998; Naas et al., 1998; Goodier et al., 2001; Sookdeo et al., 2013). The different promoter sequences regulating L1 transcription in human and mouse, and linked differences in their regulation, may likewise consequence in divergent spatiotemporal patterns of L1 expression (Faulkner and Billon, 2018). Therefore, the critical evaluation of using mice as a model to study L1 in human neurological disorders is required. Different number of retrotransposition competent L1s between human and mouse makes it more difficult and challenging to develop a model which can reliably mimic human molecular biology for L1 and its role in schizophrenia development.

Although the detailed methodology to study the L1 retrotransposition rate in neurons have been reviewed (Faulkner and Billon, 2018), but limited literature is available to dissect roles of L1 in schizophrenia and related mechanism using animal models. Manipulating neuronal sub-types with engineered L1s *in-vitro*/*in-vivo* may uphold the promise for cell-type specific investigation, although it’s a time consuming, labor-demanding and technique-challenging task.

## Conclusion

Accumulating evidence suggests that L1s prefer to re-insert in genes responsible for synapse formation in schizophrenia patients. It is warranted to explore further how L1 retrotransposition affects the synapse health chemically or physically? Does it cause hinderance in production, intracellular packaging and synaptic transmission of neurotransmitters? Moreover, currently available antipsychotics have no effects on L1 retrotransposition (Bundo et al., 2014), therefore, L1 reverse transcriptase inhibitor could be harnessed as a therapeutic choice in iPSCs derived from patients with schizophrenia and cognitively impaired mice. But the paradigm is yet to be established for proper administration of the reverse transcriptase inhibitors. Moreover, L1s preferably integrate into A/T rich region whereas nickase Cas9 can convert A into G in the target site. Therefore, nickase Cas9 would likely be a helpful tool to exploit for *in-vivo* genome correction. However, the challenge of identifying off-target editing and control still remains and further technological improvement is required. The CRISPR-based genome editing has been tested for neurological diseases like autism spectrum disorder, Alzheimer’s, epilepsy, Parkinson’s and Huntington’s diseases (Lubroth et al., 2021) which possibly heads towards clinical trials. It has not been tested for schizophrenia and is still in infancy.

Schizophrenia symptoms usually appear in adolescence and dysfunctional GABAergic signaling is the common phenotype consistently found in schizophrenia pathophysiology. As the functional maturation of GABAergic interneurons can prolongs up to post-adolescence period in primate prefrontal cortex, it is highly likely that L1s may also preferentially expressed in GABAergic interneurons. Brain has wide diversity of neuronal cells in terms of function and location, and L1’ behavior in specific cell-types needs to be explored to identify cell specific effect. Moreover, it also remains unaddressed whether the sex hormones help to trigger chromatin remodeling, L1 activation and then schizophrenia. Genome sequencing of families with schizophrenia would possibly provide a clue about the heritable pattern of L1.

Analysis of post-mortem brain of patients with schizophrenia and mouse models for schizophrenia exposed to various environmental insults at different times and exposure durations, will probably help to strengthen the loop in understanding. Maternal care in early life can modulate L1 activity, but how does its lacking translate into L1s escape and molecular pathology of schizophrenia is uncertain. There is a need to develop classification system in order to determine whether schizophrenia can be caused by L1 retrotransposition or other mechanisms? Categorization of L1s into “good or bad” and development of improved molecular reporter system to trace L1s in the genome are imperative. Thus, evidences regarding L1 retrotransposons being the cause of schizophrenia are insufficient. Further efforts are required for the development of techniques and skills to investigate the mechanism and epigenetic regulation of L1s leading to schizophrenia.
